# Sarcomatoid Carcinoma of the Ileum Mimicking a Gastrointestinal Stromal Tumor (GIST) Presenting With Primary Subfertility: A Report of a Rare Case

**DOI:** 10.7759/cureus.80716

**Published:** 2025-03-17

**Authors:** J K G Madhawa, H Wijesinghe, R A M N Rajapaksha, Vinod Saranga, Vihanga Chamod Wickramasinghe

**Affiliations:** 1 Department of Surgery, Colombo South Teaching Hospital, Colombo, LKA; 2 Department of Pathology, Faculty of Medicine, University of Colombo, Colombo, LKA; 3 Department Surgery, Karapitiya National Hospital, Galle, LKA

**Keywords:** ileal tumor, peritoneal metastases, rare cancer, sarcomatoid carcinoma, small intestine cancer

## Abstract

Sarcomatoid carcinoma (SCA) is a rare and aggressive malignancy characterized by the coexistence of epithelial and mesenchymal components. While it has been described in various organs, SCA of the ileum is exceptionally rare, with only handful of cases reported in the literature to date. We report a case of a 38-year-old woman presenting with primary subfertility and episodic lower abdominal pain, initially attributed to possible adenomyosis. During subfertility evaluation with a laparoscopic dye test, an incidental polypoidal growth was detected in the distal ileum. Contrast-enhanced computed tomography (CECT) revealed a heterogeneously enhancing mass in the right iliac fossa, suspected to be a gastrointestinal stromal tumor (GIST). Following multidisciplinary team discussions, surgical excision was performed with a laparoscopic assisted right hemicolectomy. Histopathological analysis confirmed a biphasic tumor with carcinomatous and sarcomatous components, consistent with SCA. Evaluation with immunohistochemical markers further narrowed down differential diagnoses. Postoperatively, the patient developed metastatic progression, as evidenced by peritoneal masses and an abdominal wall deposit on repeat CECT. She subsequently underwent adjuvant chemotherapy. SCA of the ileum poses significant diagnostic challenges due to nonspecific clinical features and its rarity. This case underscores the diagnostic complexities and therapeutic challenges of ileal SCA. Collaborative research is essential to develop effective treatment strategies for this rare malignancy.

## Introduction

Primary sarcomatoid carcinoma (SCA) is a rare and aggressive malignancy in which both epithelial (carcinomatous) and mesenchymal (sarcomatous) components coexist in the same tumor mass. SCA has been reported in various organs commonly lungs, uterus, salivary glands, thyroid glands, gallbladder, stomach, and esophagus [1-3]. They are very rarely found in small intestine, and only handful of cases have been reported in literature. To our knowledge, only 14 cases of primary SCA of the ileum have been reported in the literature up to now [4]. Primary SCA of the small intestine often presents late due to nonspecific symptoms, and inaccessibility via routine endoscopy delays its diagnosis. It carries an overall poor prognosis with a median survival rate of just a few months and a five-year survival rate as low as 20% [5]. SCA was first described in the small intestine using the term enteroblastoma back in 1973. Other terms, such as carcinosarcoma, metaplastic carcinoma, and spindle cell carcinoma, were subsequently used in other organs, reflecting the ambiguity of its origin [6]. At present, SCA is the widely accepted term used in the literature.

## Case presentation

**Figure 1:**
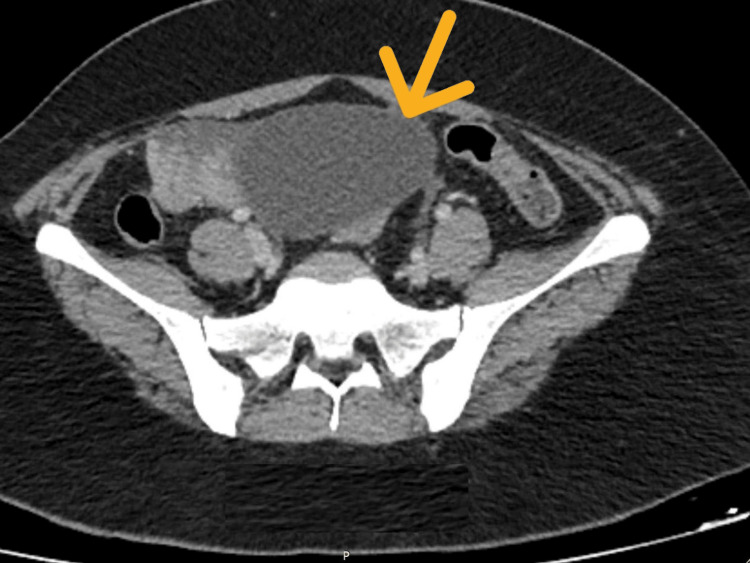
Craniocaudal cross-section of the distal ileal lesion (yellow arrow) on preoperative CECT. Arrow is pointed at the SCA of the ileum. The tumor appears hypodense relative to surrounding soft tissue. CECT: contrast-enhanced computed tomography

Here, we report a case of a 38-year-old woman who was referred by the gynecology team for a polypoidal growth detected in the ileum, during a laparoscopic dye test done for the evaluation of primary subfertility. She had been having episodic abdominal pain mainly in the lower abdomen for four to five months. She denied any other gastrointestinal symptoms. Abdominal examination revealed suprapubic and right iliac fossa tenderness, but no palpable masses were detected. Blood tests did not reveal a significant anemia. Her hemoglobin was 12.1g/dL (11.0-14g/dL) and the Carcinoembrayonic antigen (CEA) level was within normal limits. Other biochemical and hematological tests were unremarkable. Contrast-enhanced computed tomography (CECT) of the chest, abdomen, and pelvis showed a heterogeneously enhancing soft tissue mass in the right iliac fossa, measuring 5.1 cm × 5.6 cm × 4.9 cm without any evidence of nodal or distant metastasis. The radiological features were suggestive of a a gastrointestinal stromal tumor (GIST) of the distal ileum (Figure 1). An arcuate uterus with myometrial adenomyosis was also present.

**Figure 2:**
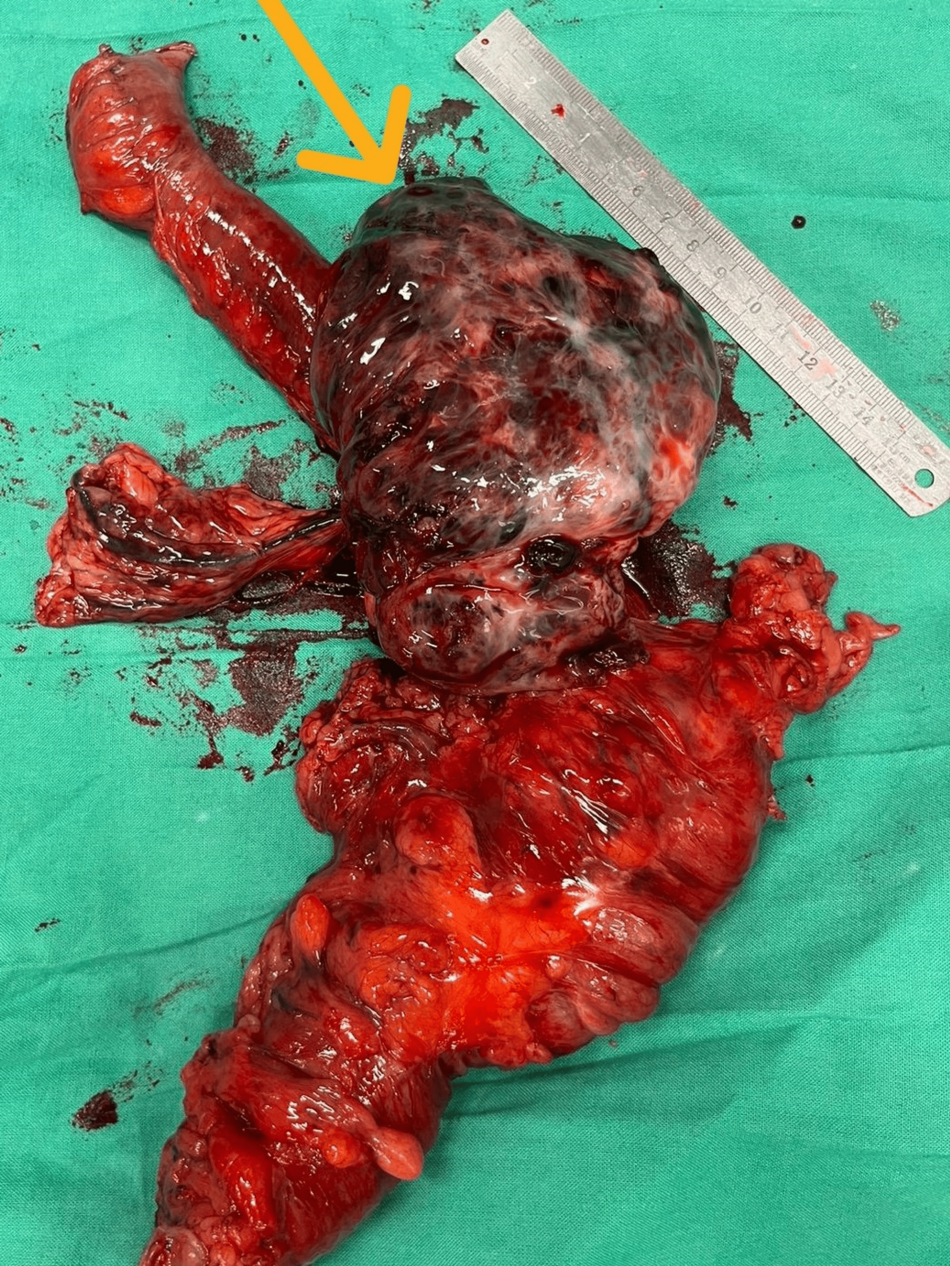
Postoperative specimen of the distal ileal tumor (yellow arrow) Arrow pointing at the ileal lesion in the resected specimen. Macroscopically, the tumor appears to have bluish-black discoloration measuring 13 cm x 8 cm x 7 cm, and its consistency was hard.

Surgical resection of the tumor was planned following multidisciplinary team (MDT) discussion, and a laparoscopic-assisted right hemicolectomy was carried out three weeks after the CECT. Unexpectedly, during the surgery, it was noted that tumor had a significant interval enlargement following the CECT. It had rapidly progressed to a lesion of around 13 cm x 8 cm x 7 cm (Figure 2). Visual inspection of the rest of the bowel peritoneum and the liver was unremarkable. The tumor along with ileum and right colon were exteriorized via a lower midline laparotomy incision. The affected bowel segment was resected, and an end-to-side hand-sewn ileo-colic anastomosis was created using 3/0 polyglactin in interrupted sero-submucosal pattern. Postoperative recovery was uneventful, and due to the laparoscopic approach, postoperative pain was minimal and early enteral feeding could be established. She was discharged from hospital after five days.

**Figure 3:**
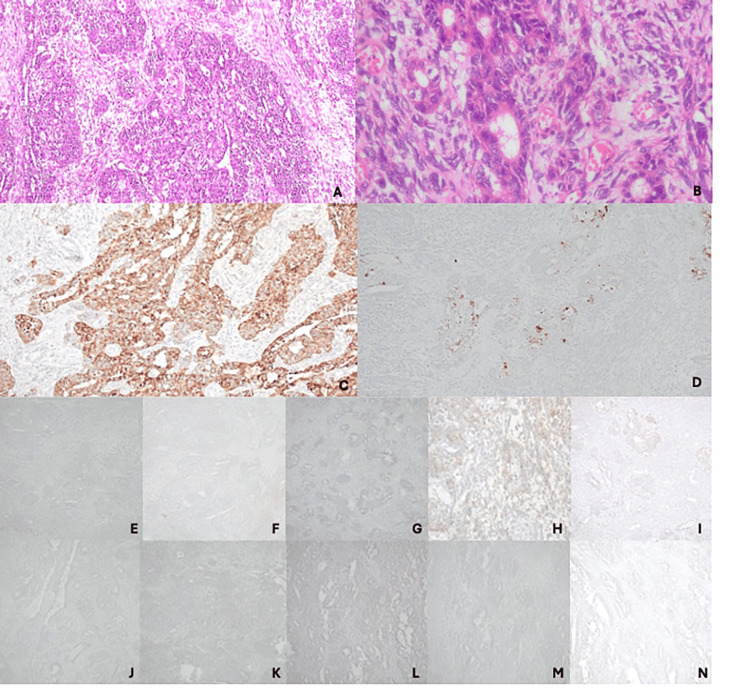
(A) The tumor was composed of epithelial and sarcomatous components (H&E, x100). (B) The epithelial component was composed of cribriform glands lined by pleomorphic cells with vesicular nuclei and eosinophilic cytoplasm. The sarcomatous component comprised spindle cells with plump vesicular nuclei (H&E, x400). (C) The epithelial component showed diffused staining for AE1/AE3 with focal staining in the sarcomatous component (AE1/AE3, x100). (D) There was focal staining for CK7 in the epithelial component (CK7, x20). The tumor was negative for (E) CK20, (F) synaptophysin (focal non-specific staining), (G) chromogranin, (H) CD99 (weak non-specific staining), (I) bcl 2, (J) TLE1, (K) calretinin, (L) SAL4, (M) PAX 8, and (N) GATA3 H&E: hematoxylin and eosin

The resected specimen unveiled an irregular friable growth with solid and cystic areas and areas of hemorrhage. The preliminary histopathology report revealed a biphasic malignant tumor with epithelial and mesenchymal components and clear resection margins. The epithelial component was composed of glandular structures and cribriform nests lined by columnar cells with pleomorphic vesicular nuclei and eosinophilic cytoplasm (Figure 3). The mesenchymal component comprised spindle-shaped cells with elongated plump vesicular nuclei with scanty eosinophilic cytoplasm (Figure 3). Mitoses were seen in both epithelial and mesenchymal elements. Malignant heterologous elements were not seen. Both epithelial (diffuse) and mesenchymal(focal) components stained positively with AE1/AE3 (pancytokeratin) and CK20 was negative (Figure 3). The epithelial component showed focal staining with CK7 and nonspecific staining with synaptophysin and CD99. Chromogranin was negative, excluding the presence of neuroendocrine differentiation. Negativity for calretinin excluded a biphasic mesothelioma, negativity for SALL4 excluded a germ cell tumor, and negative staining with TLE1 and BCL2 had excluded the possibility of synovial sarcoma (Figure 3). Negativity for metastatic deposits from the female genital tract and breast were unlikely due to negativity for PAX8 and GATA3, respectively (Figure 3). Based on the morphological and immunohistochemical findings, a final diagnosis of SCA was made.

**Figure 4:**
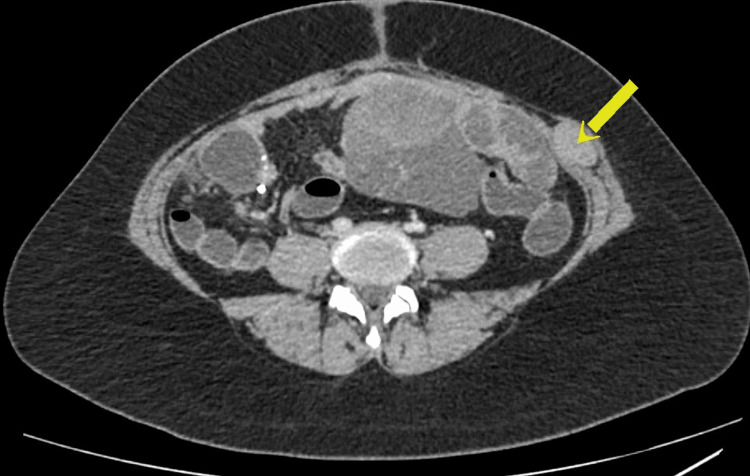
Craniocaudal view of the postoperative CECT with lateral abdominal wall metastases (yellow arrow). Arrow pointing at one of the suspected abdominal wall metastatic lesions. Lesion is positioned on the left lateral abdominal wall, and it exhibits a similar density to the surrounding soft tissue. CECT: contrast-enhanced computed tomography

During the follow-up clinic visit after three weeks, she complained of residual episodic abdominal pain mainly in the suprapubic and umbilical region. Clinical evaluation revealed tenderness over the specific region. The possibility of a residual collection or infection was ruled out with an unremarkable white blood cell count, C-reactive protein levels, and negative imaging with ultrasound abdomen. Due to persistent episodic pain, she underwent a repeat CECT of abdomen, which unfortunately revealed disease progression with multiple large peritoneal masses and a left lateral abdominal wall mass representing metastatic deposits from the known malignant bowel tumor (Figure 4). Following MDT discussion, she was directed for chemotherapy under a clinical oncologist and has currently completed two cycles of chemotherapy.

## Discussion

Adenocarcinomas, neuroendocrine tumors, sarcomas, and lymphomas are the four most prevalent types of malignant tumors that originate in the small intestine [4]. Jejunal primary SCAs are the commonest recorded in the literature, followed by that of ileal and duodenum [4]. Patients commonly present with anemia, abdominal pain, abdominal masses gastrointestinal masses, and weight loss [5]. Although our patient had abdominal pain, the concurrent history of subfertility had diluted its significance, and abdominal pain was attributed to be a part of the spectrum of subfertility secondary to adenomyosis. This may have contributed to the delay in diagnosis. Since the tumor was positioned in the distal ileum, endoscopic retrieval of a tissue sample of the tumor was a practical difficulty.

Based on the radiological features, the lesion of interest in the ileum was suspected to be a GIST, but the presence of malignant epithelial and mesenchymal tissue and cytokeratin positivity on histological examination of the resected specimen confirmed the diagnosis of SCA. This highlights the potential limitation of CECT as a sensitive imaging modality to diagnose SCA. There are no established treatment guidelines for SCA; however, surgical excision of the tumor remains the primary treatment objective [6]. The diagnosis of SCA was based on based on pathological observations and supported by immunohistochemical staining. A panel of immunohistochemical markers were utilized to exclude the differentials, which included biphasic mesothelioma (calretinin), germ cell tumor (SALL4), biphasic synovial carcinoma (CD99, bcl - 2, TLE1), and a metastatic deposit of a carcinosarcoma/metaplastic carcinoma from the female genital tract (PAX8) and breast (GATA 3). Further specific genetic studies for SS18-SSX1/2/4 fusion gene were recommended for further exclusion of synovial sarcoma.

SCA may exhibit either a monophasic or biphasic pattern [6]. The monophasic pattern is typically characterized by a predominance of mesenchymal-like components with minimal to absent epithelioid areas. By contrast, biphasic tumors feature a combination of epithelial-like and mesenchymal-like cells as in this case [6].

While >50% of patients with SCA in the literature had lymphovascular invasion by the time of presentation [5], our patient did not have any clinical or radiological evidence of metastasis at the time of initial surgery The most common metastatic locations are the lung, distant lymph nodes and liver, while the brain and pelvic bones may also be involved [6]. Due its aggressive nature median survival after the diagnosis of a small bowel SCA is around eight to nine months [6]. At present, there are no standardized guidelines for adjuvant chemotherapy or radiotherapy in the treatment of SCA [6]. Evidence does not support either chemotherapy or radiotherapy to improve the long-term survival rate [7]. In this case, the patient underwent surgical excision of the ileal lesion followed by adjuvant chemotherapy.

The data on SCAs of the ileum reported on the literature (Table 1) summarize patient demographics, tumor characteristics, immunohistochemical markers, and survival outcomes. The patients range in age from 44 to 76 years, with a mean age of approximately 59 years, and there is a slight male predominance (66.6%), as nine of the 14 cases are male. Tumors present in various morphologies, including polypoid, endophytic, ulcerating, and nodular forms. The maximum tumor size varies significantly, ranging from 3 cm to 15 cm, with many cases reporting sizes between 4.5 cm and 9.2 cm. Some cases involve single lesions, while the lesion count is unspecified in others. Immunohistochemical analysis shows that cytokeratin (CK) positivity is frequently observed (66.6%), indicating epithelial differentiation, while vimentin positivity present in all reported cases, reflects mesenchymal differentiation. However, some cases lack complete immunohistochemical data. Survival outcomes are generally poor, with overall survival ranging from just 0.2 months to 39 months, and a median survival of less than a year. Metastasis is noted in approximately half of the cases, contributing significantly to the dismal prognosis. These findings underscore the aggressive nature of ileal SCAs and highlight the critical need for improved diagnostic and therapeutic approaches.

**Table 1:** Summary of the diagnostic information of SCA of the ileum reviewed in this article ID: identification number, SCA: sarcomatoid carcinoma, CA: carcinoma, N/A: not available, CK: cytokeratin, OS: overall survival. * - Patients who were alive at the time of publication.

ID	Age	Gender	Diagnosis	No. of lesion(s)	Maximal diameter (cm)	Morphology	Metastasis	CK	Vimentin	OS (months)	Reference
1	44	M	Enteroblastoma	1	N/A	Polypoid	Yes	N/A	N/A	N/A	[8]
2	54	F	Anaplastic and SCA	1	4.5	Endophytic	No	-	N/A	12*	[9]
3	62	M	Anaplastic and SCA	1	5	Endophytic	Yes	-	N/A	20	[9]
4	45	M	Pleomorphic CA	1	3	Endophytic	No	+	+	0.2	[10]
5	57	M	Pleomorphic CA	1	14	Endophytic	No	+	+	6*	[10]
6	63	M	Pleomorphic CA	1	6	Endophytic	No	+	+	39*	[10]
7	68	F	SCA	1	N/A	N/A	No	N/A	N/A	N/A	[11]
8	75	M	SCA	1	N/A	N/A	No	+	+	N/A	[11]
9	76	F	SCA	1	5	Ulcerating	N/A	+	+	2	[12]
10	53	M	Anaplastic and SCA	N/A	N/A	Polypoid	Yes	+	+	N/A	[13]
11	56	M	SCA	1	9.2	Ulcerating	Yes	+	+	3	[14]
12	62	M	SCA	1	15	Ulcerating	No	+	+	3*	[15]
13	60	M	N/A	N/A	N/A	Nodular	Yes	+	N/A	N/A	[16]
14	58	F	SCA	1	3	Polypoid	No	+	+	0.36	[17]
15	38	F	SCA	1	10	Polypoid	Yes	+	N/A	4*	This case

## Conclusions

SCA of the ileum is an exceedingly rare and aggressive malignancy presenting with nonspecific clinical features, often delaying diagnosis and treatment. Surgical resection remains the mainstay of management, while the role of adjuvant therapy remains undefined. Despite initial locoregional disease, the rapid progression to metastatic deposits emphasizes the poor prognosis associated with SCA and the need for further collaborative research to establish effective treatment guidelines.
